# Association between abnormal lipid metabolism and tumor

**DOI:** 10.3389/fendo.2023.1134154

**Published:** 2023-05-25

**Authors:** Chunyu Li, Fei Wang, Lili Cui, Shaoxin Li, Junyu Zhao, Lin Liao

**Affiliations:** ^1^ Department of Endocrinology and Metabology, The First Affiliated Hospital of Shandong First Medical University and Shandong Provincial Qianfoshan Hospital, Shandong First Medical University, Shandong Key Laboratory of Rheumatic Disease and Translational Medicine, Shandong Institute of Nephrology, Jinan, China; ^2^ Department of Endocrinology and Metabology, Shandong Provincial Qianfoshan Hospital, Cheeloo College of Medicine, Shandong University, Jinan, China

**Keywords:** lipid metabolism, tumor, sphingolipid, cholesterol, therapy

## Abstract

Metabolic Reprogramming is a sign of tumor, and as one of the three major substances metabolism, lipid has an obvious impact. Abnormal lipid metabolism is related to the occurrence of various diseases, and the proportion of people with abnormal lipid metabolism is increasing year by year. Lipid metabolism is involved in the occurrence, development, invasion, and metastasis of tumors by regulating various oncogenic signal pathways. The differences in lipid metabolism among different tumors are related to various factors such as tumor origin, regulation of lipid metabolism pathways, and diet. This article reviews the synthesis and regulatory pathways of lipids, as well as the research progress on cholesterol, triglycerides, sphingolipids, lipid related lipid rafts, adipocytes, lipid droplets, and lipid-lowering drugs in relation to tumors and their drug resistance. It also points out the limitations of current research and potential tumor treatment targets and drugs in the lipid metabolism pathway. Research and intervention on lipid metabolism abnormalities may provide new ideas for the treatment and survival prognosis of tumors.

## 1 Introduction

In the past two decades, due to urbanization, economic growth and population aging, the world has experienced a rapid epidemiological transformation. Infectious diseases have been largely replaced by cardiovascular diseases as the leading cause of death, and dyslipidemia is the second largest risk factor related to cardiovascular disease, which is often ignored. Previous epidemiological studies have stated a positive correlation between abnormal lipid metabolism and increased risk of atherosclerosis, chronic kidney disease, Alzheimer’s disease and osteoporosis. Furthermore, excessive lipid accumulation is also associated with some other diseases, including diabetes (1), non-alcoholic fatty liver disease/hepatitis, pituitary dysfunction, testosterone deficiency and hypothyroidism (2). At the same time, massive studies have also reported the role of lipid metabolism in regulating the biological process of tumors, especially on tumorigenic signal pathway, iron death and tumor microenvironment (3).

Alterations in energy metabolism is deemed to be a denote of cancer and an important target for cancer treatment. Uncontrolled and unlimited cell proliferation of cancer cells requires efficient energy sources, and in order to meet the needs of cancer cells, lipid metabolism is over-activated (4). Therefore, metabolic abnormalities, including abnormal lipid metabolism, occurred in the whole process of tumorigenesis and progression. Study on the mechanism of abnormal lipid metabolism in tumor can provide new ideas for tumor treatment.

**Figure 1:**
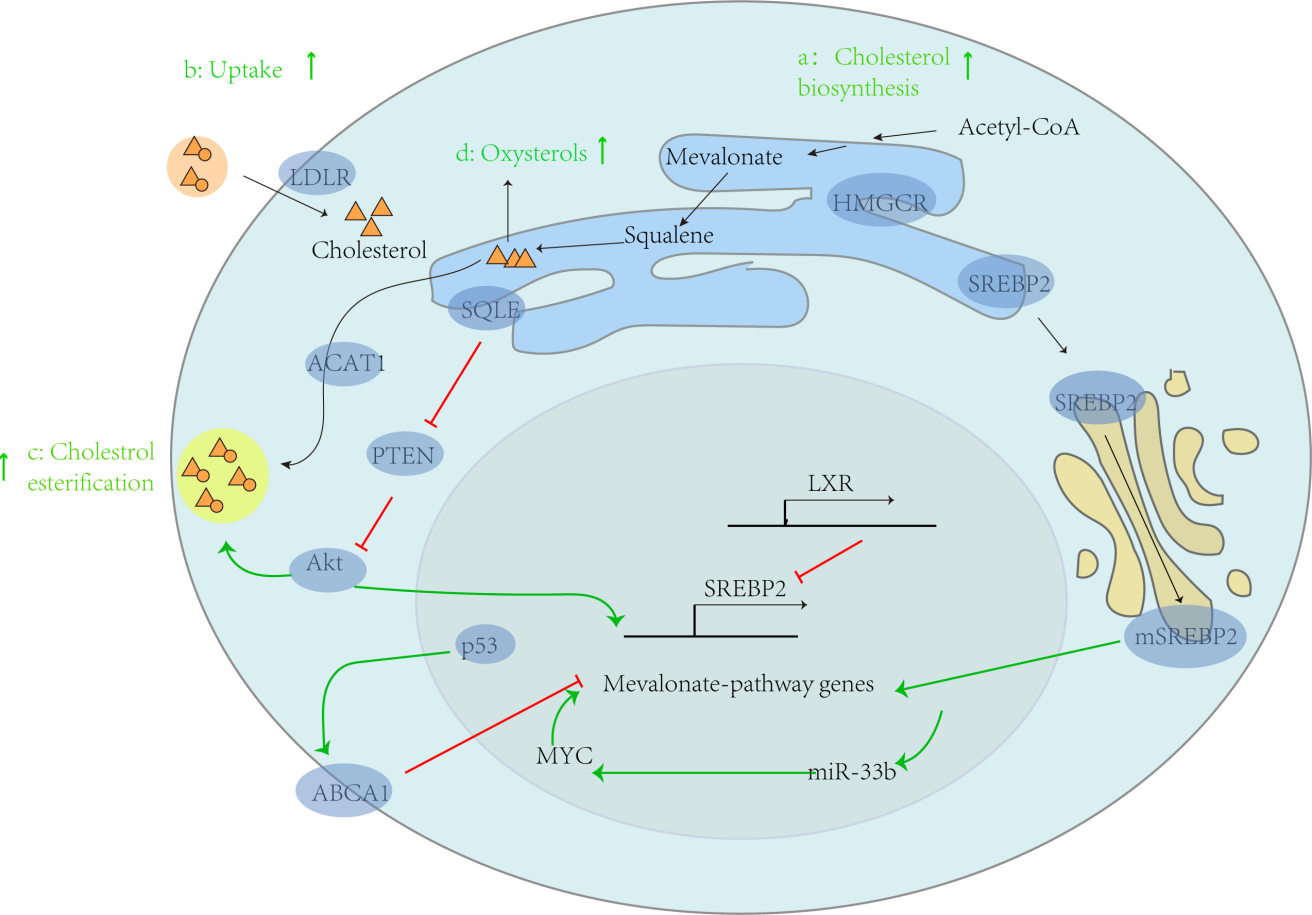
Abnormal lipid metabolism and tumor progression. In tumor cells, cholesterol metabolism is generally enhanced, thereby supporting the progression of cancer. This can be demonstrated from four aspects: a: enhanced cholesterol biosynthesis, b: increased exogenous cholesterol uptake by LDLR, c: increased cholesterol esterification by ACAT1, and d: increased production of hydroxysterols. In addition, the intrinsic driving factors of its carcinogenesis include: (1) activating carcinogenic genes such as MYC, which leads to the activation of mevalonate pathway genes, further increasing the expression of miR-33b, thus increasing the expression of MYC through positive feedback; (2) In the process of relying on target ABCA1, p53 mediated inhibition of the mevalonate pathway is absent; (3) SQLE activates Akt by inhibiting PTEN expression, leading to CE accumulation.

Lipid metabolism pathway plays an important role in many metabolic pathways. Lipids are defined as a diverse group of molecules insoluble in water, including triacylglycerides, phosphoglycerides, sterols and spatholipids. Among them, fatty acid is the principal component of triacylglycerol ester, which is mainly used for energy storage. Phosphoglycerides, along with sterols and sphingolipids, are the main structural components of biofilms. In addition, lipids can also play an important role as second messengers and hormones in signal transmission (5). Changes in lipid metabolism can alter the stability of biofilms and are associated with tumor aggressiveness. Many tumor cells exhibit a high rate of *de novo* lipid synthesis. In 1953, some scholars proposed that tumor tissues could synthesize lipids in a manner similar to embryonic tissues (6). Therefore, there is increasing evidence that cancer cells exhibit specific changes in different aspects of lipid metabolism and may be involved in the disease progression of tumors (**Figure 1**). This review mainly introduces the research of abnormal lipid metabolism and tumor and the potential role of drugs that related to lipid metabolism in tumor from three aspects: cholesterol, triglyceride and Sphingolipid.

## 2 Abnormal cholesterol metabolism and tumor

### 2.1 Transcription regulatory factors related to cholesterol homeostasis

Sterol regulatory element binding proteins (SREBPs), liver X receptors (LXRs) and nuclear factor erythroid 2 related factor-1 (NRF1) are the main transcription regulators regulating cholesterol homeostasis (7).

SREBPs can affect cholesterol synthesis by regulating the activity of HMGCR. Some studies have found that the inhibition of HMGCR is significantly related to the reduction of incidence rate of epithelial ovarian cancer (8). However, this finding does not indicate that drugs that inhibit HMGCR can reduce their risk of disease, and further research is needed to understand whether similar associations exist with these drugs. In addition, it was found that SREBP1c and SREBP-2 were not overactivated in mouse tumors after injection of diethylnitrosamine to induce hepatocellular carcinoma (9). Therefore, further in-depth research is needed on the role of SREBPs in tumor cells. Another key enzyme for cholesterol synthesis, squalene epoxidase (SQLE), is also regulated by SREBPs. The SQLE transcription gene can inhibit PTEN protein expression, activate Akt signaling, and lead to cholesterol ester accumulation (**Figure 1**). A study by the Chinese University of Hong Kong confirmed that SQLE can promote the proliferation of colorectal cancer (CRC) cells by inducing cell cycle progression and inhibiting apoptosis, and confirmed that the SQLE inhibitor terbinafine can inhibit the growth of CRC through synergistic effects with oxaliplatin and 5-fluorouracil (10).

LXRs are nuclear receptors encoded by the gene nr1h3 (LXR-α) and nr1h2 (LXR-β), some functional oxysterols, such as 24-hydroxycholesterol (HC), 25-HC and 27-HC, are important activators of LXRs. In breast cancer, LXR-α Cancer cell lines with high mRNA expression are more sensitive to the inhibition of 22 (R)-HC induced (11), and estrogen receptor status is negatively correlated with the ability of LXR to induce typical target gene expression, that is, LXR in breast cancer cells with negative estrogen receptor has a stronger ability to induce typical target gene expression (12).

NRF1 can also affect cholesterol homeostasis by influencing the above two regulatory factors. When cholesterol concentration increases, it blocks the nuclear translocation of NRF1 and inhibits its inhibition of the LXR pathway. NRF1 can also regulate the activation of SREBPs and lipid metabolism by regulating of endoplasmic reticulum transmembrane protein 33 (10).

### 2.2 Abnormal cholesterol metabolism and carcinogenic signal pathways

In the tumor microenvironment, the endogenous and exogenous signals of cells can reprogram cholesterol metabolism, thus promoting the occurrence of tumors (13). The following mainly introduces the carcinogenic signal pathways related to abnormal cholesterol metabolism.

The activation of PI3K/AKT/mTOR signal can upregulate intracellular cholesterol levels (14), which is an important pathway for regulating cell cycle and is directly related to cell dormancy, proliferation, carcinogenesis, and lifespan. Methoxyvalerate can activate PI3K, mTOR, NF-κB, and inhibit the transcription of genes such as P21 and P27 (15), leading to changes in apoptosis, cycle, autophagy, and migration of cancer cells (13). Hanai et al. (16) reported that ATP citrate lyase (ACL) exerts anti-tumor effects by downregulating the PI3K/AKT pathway. Studies have also revealed a CRC liver metastasis specific cholesterol metabolism pathway: hepatocyte growth factors from the liver environment activate the c-Met/PI3K/AKT/mTOR axis in CRC cells to activate the SREBP2 dependent cholesterol biosynthesis pathway (17).

Lysosomal cholesterol can inhibit mTORC1 signal through the SLC38A9 Niemann Pick C1 (NPC1) complex (13). Studies have shown that the deletion of the NPC1 gene leads to cholesterol accumulation in lysosomes, leading to excessive activation of mTORC1, impaired mitochondrial function, and neurodegeneration (18). In addition, Niemann Pick C1 like 1 gene knockout has been shown to prevent the occurrence of colitis related cancer (14). MTORC1 may become a new target for inhibiting tumor progression (18).

Mevalonate can further activate Hippo signal by activating the TP53/SREBP pathway in cancer cells (15). P53 is a key tumor suppressor factor that maintains metabolic homeostasis and coordinates cellular stress responses. The p53 and Hippo pathways collaborate at multiple levels to fine-tune SREBP activity and regulate cholesterol/lipid levels (19). The TP53 gene change is often observed in poorly differentiated thyroid cancer and undifferentiated thyroid cancer, which may lead to the disruption of cell cycle checkpoints and DNA repair mechanisms, leading to the tolerance of tumor cells to cumulative genetic instability (20). It was found that TP53 mediated activation of cholesterol synthesis *via* SREBP pathway can induce breast cancer cell proliferation and self-renewal through preacylation of GTPases (14).

The Hedgehog pathway is a classic cancer-related signal pathway controlled by G-protein coupled receptors, known as Smoothened receptors. It has been reported that cholesterol could activate cancerigenic Hedgehog signaling by binding to Smoothened receptors at first hand (21). Cholesterol can also covalently modify proteins, including smoothened and hedgehog, and promote the formation of specialized membrane microdomains to regulate the signal pathway of tumorgenesis and cancer progression (7).

Furthermore, cholesterol specifically binds to the PDZ domain of scaffold proteins, for example, the N-terminal PDZ domain of NHERF1/EBP50. NHERF1/EBP50 is a master regulator of oncogenic signal networks that is involved in cell proliferation and tumor formation by assembling cancer-associated proteins, including those belonging to the PI3K/Akt and Wnt/β-catenin pathways (15).

RTK/Ras signal is a major regulatory gene that induces the synthesis of cholesterol and leads to intracellular cholesterol accumulation by activating the SREBP transcription factor, mutations and activation of which are common in childhood acute myeloid leukemia (14).

In summary, cholesterol is mainly regulated by transcription factors such as SREBPs, LXRs, NRF1, and there are also connections between these regulatory factors. Importantly, cholesterol metabolism can affect multiple carcinogenic pathways, including PI3K/AKT/mTOR, NF-κB,WNT/β-catenin, Hedgehog, TP53/SREBP, NPC1/mTOR, RTK/Ras, and other signal pathways related to regulating tumor cell proliferation, growth, and angiogenesis. Some transcription factors related to tumors are also affected by cholesterol metabolism. In summary, cholesterol metabolism, including cholesterol regulatory factors, is closely related to tumor carcinogenic signal pathways and promotes tumor progression, potentially becoming a key target for tumor treatment.

### 2.3 Epigenetics promotes tumor progression by influencing lipid metabolism, including cholesterol metabolism

It is worth noting that lipid metabolism is also regulated by epigenetic imprinting. It includes DNA methylation, post translation histone modification and non-coding RNA.

#### 2.3.1 DNA methylation

DNA methylation is the addition of a methyl group to cytosine in dinucleotide CpG mediated by DNA methyltransferase (DNMT), and S-adenosylmethionine is the methyl donor. Compared with normal tissues, tumor cells are characterized by increased (hypermethylation) and decreased (hypomethylation) DNA methylation. Hypermethylation is usually localized and can occur within regulatory elements, leading to gene silencing involved in various cellular processes, such as apoptosis, cell cycle, and DNA repair genes, many of which are tumor suppressor genes. In contrast, hypomethylation is usually holistic and affects large genomic domains, which are associated with chromosomal instability, activation of transposable factors and oncogenes, and loss of genomic imprinting (22). DNA methylation can promote tumor progression by influencing lipid metabolism. Firstly, methylation can affect tumor progression, and methylation is involved in various metabolic pathways including fatty acids, acetyl CoA, tricarboxylic acid cycle, phospholipid metabolism, etc. The possible mechanism is that they participate in lipid metabolism by regulating cell metabolic transcription factors and tumor suppressor p53. Chen L et al. have demonstrated that chromatin modified lymphoid-specific helicase (LSH) lead to the deubiquitination of p53 in a pyruvate kinase dependent manner, promote p53 transcription activity and p53 mediated lipid metabolism, and affect the expression of a variety of lipid metabolism related genes. For example, carnitine palmitoyltransferase 1B, apolipoprotein B mRNA editing enzyme, catalytic peptide, cytochrome P450, et al. (23). In addition, Li Z et al. found that the methylation of Retinoic Acid-inducible Gene-I mediated by the demethylase JMJD4 inhibits HMGCR phosphorylation by binding to AMPK, thereby improving the activity of HMGCR enzyme and cholesterol synthesis (24). It is worth noting that hypermethylation is also involved in lipid regulation to a certain extent. The hypermethylation of the 6 promoters of the 27 members of the solute carrier family promotes the proliferation of nasopharyngeal carcinoma by regulating lipid metabolism but inhibits its metastasis (25). At present, demethylating agents, such as DNMT inhibitor Decitabine, have been proven to be effective in treating cancer such as myelodysplastic syndrome and acute myeloid leukemia (26). Low dose Decitabine can increase the sensitivity of cervical cancer cells to chemotherapy (27). DNMT inhibitors also showed anti-tumor activity in animal models of PCa (22), but clinical trials did not show significant effects.

Methylation affects a variety of tumor progression, including but not limited to nasopharyngeal carcinoma, breast cancer, liver cancer, endometrial cancer, prostate cancer, but the research on the relationship between methylation and lipid metabolism is not complete at present, and more evidence is needed to reveal its internal relationship. Further research is needed to evaluate the clinical application value of demethylating agents alone or in combination with other drugs as treatment options for prostate cancer or other demethylated tumors.

#### 2.3.2 Histone modification

Histone modification is also closely related to lipid metabolism, and can affect its metabolism by regulating cholesterol related regulatory factors and metabolic intermediates. Histone methyltransferase KMT5A regulates the occurrence of papillary thyroid cancer *in vitro* and the expression of lipid metabolism molecules such as SREBP1, SCD (catalytic saturated fatty acids), FASN (fatty acid synthase), and ACC (acetyl CoA carboxylase) (28). Obesity related factors induce ATP citrate lyase nuclear transfer, which enhances pyrimidine metabolism by regulating histone acetylation in endometrial cancer (29). In addition, in non-alcoholic fatty liver disease mouse cells, histone deacetylase SIRT6 inhibits liver disease progression by promoting fatty acid oxidation and regulating metabolic related gene expression (30). It is worth noting that Tonini C et al. found that in the HepG2 human liver cell culture model, an epigenetic reader - bromine domain and extracellular domain (BET) protein can reduce lipid content by blocking the expression and processing of SREBP-2, resulting in reduced expression of target genes involved in lipid biosynthesis, uptake, and intracellular transport (31). In prostate cancer, SPOP gene mutations can induce ubiquitination and proteasome degradation of BET protein by recognizing the degron motif, increase the expression of cholesterol synthesis genes, and activate the AKT-mTORC1 pathway (32). This may indicate that BET inhibitors have the potential to become a new anticancer therapy in liver and prostate cancer. In addition, Lin Z et al. found that quinazoline histone deacetylase inhibitors affect cholesterol biosynthesis and gene expression pathways related to mevalonate in prostate cancer cells, becoming a new hope for the treatment of prostate cancer (33). Although the histone deacetylase inhibitor butyrate has shown positive results in reducing cholesterol in animal studies, it has not yet been confirmed in clinical studies (34). Its potential mechanism may be to reduce the activity of SREBP-2, but it does not increase the expression of LDLR and HMGCR involved in cholesterol uptake, and butyrate can also indirectly affect the expression of glucagon peptide 1 to affect cholesterol levels (34). So, it is uncertain whether butyrate affects blood lipid levels through SREBP-2 activity. In summary, de histone modification is expected to become a potential tumor target.

#### 2.3.3 Non-coding RNA

In recent years, non-coding RNA has been well studied in various diseases, especially in tumor research. Among them, Ni Y et al. found that non-coding RNA 15a-5p can significantly inhibit the fatty acid synthesis of cancer cells by inhibiting acetate uptake. And a new mechanism was proposed that miR-15a-5p inhibits lipid metabolism by inhibiting ACSS2 mediated acetyl CoA activity and histone acetylation, thereby inhibiting cancer cell metastasis (35).

Metabolic and epigenetic changes are the characteristics of cancer cells. There is no doubt about the interaction between lipid metabolism and epigenetics. Epigenetics, including methylation, histone modification and non-coding RNA, is related to the level of lipid metabolism and regulation of lipid metabolism, including demethylating agent dicidibine, histone methyltransferase KMT5A, histone deacetylase SIRT6, BET inhibitor Histone deacetylase inhibitors such as quinazoline exhibit anti-tumor activity. At present, the mechanisms by which epigenetics affect lipid metabolism include regulating the activity of transcription factors P53 and SREBP-2. Although it has not been confirmed yet, it is indeed a promising new anticancer therapy.

### 2.4 The important role of low-density lipoprotein and high-density lipoprotein in cholesterol metabolism

Hypercholesterolemia can promote tumor growth, especially with an increase in low-density lipoprotein (LDL) (36). Epidemiological studies have shown that LDL and ox-LDL are closely related to breast cancer, colorectal cancer, pancreatic cancer and other malignant tumors, suggesting that LDL and ox-LDL play an important role in the occurrence and development of cancer (37). Recently, it has been found that LDLR is overexpressed in various cancers, such as hepatocellular carcinoma, breast cancer, colorectal cancer, prostate cancer, etc. Due to the fact that cancer cells require more cholesterol to obtain energy than normal cells, they may increase cholesterol levels through receptor-mediated endocytosis of LDL (38). Abnormal lipid metabolism can lead to lipid toxicity, leading to oxidative stress and significantly increasing levels of reactive oxygen species (ROS). The gradual increase in oxidative stress can lead to the oxidation of intracellular LDL to ox-LDL. Ox-LDL binds to lectin like oxidized low-density lipoprotein receptor-1 (LOX-1) and Cluster Differentiating 36 (CD36), inducing mutations, leading to inflammation, cell proliferation, and cancer metastasis. In addition, oxidative stress promotes DNA damage in cancer, leading to malignant transformation and carcinogenesis (39). Elevated plasma ox-LDL was detected in breast cancer, gastric cancer and colon cancer (36). Yang L et al. also demonstrated that oxidized low-density lipoprotein induced by hypercholesterolemia can promote the generation of bladder cancer stem cells through the CD36/STAT3 signal axis (40). Due to the complex roles of LDL and ox-LDL in cancer, further research is needed to investigate the potential therapeutic effects of lipid metabolism in cancer treatment.

Some observational study have found that the reduction of high-density lipoprotein cholesterol (HDL-C) level is also related to some cancer types, such as CRC, lung cancer, breast cancer, ovarian cancer, thyroid cancer and gastric cancer (41). However, whether the decrease in HDL-C levels is a consequence or causal factor in the development and progression of cancer remains a controversial issue. Breast cancer patients have low levels of HDL-C, so some people think that low levels of HDL-C may be a risk factor for breast cancer (42). In addition, low HDL-C levels are associated with a high risk of gastric cancer, high lymphatic and vascular infiltration rates, advanced lymph node metastasis, and poor prognosis (33). Ganjali S et al. also proposed that low levels of HDL-C are significantly associated with cancer mortality (39). There are also studies analyzing that malignant cells consuming higher cholesterol to promote tumor growth may lead to plasma cholesterol depletion (43), which may be the reason for the above research results. However, some studies have found that SR-B1-mediated HDL uptake can enhance cell proliferation in breast cancer, prostate cancer and ovarian cancer, but these effects may be cancelled by the antioxidant and anti-inflammatory properties of HDL (4). Some scholars also believe that cancer type may be an important factor affecting the relationship between HDL-C and cancer (41), but the specific mechanism may require further research.

Obviously, the relationship between cholesterol and cancer may not be a simple dual factor relationship, and there may be a potential conditional factor that can reverse the relationship between cholesterol and cancer progression. One possible conditional factor is the tissue origin of cancer, where different tissues have different cholesterol requirements and composition ratios. Another possible conditional factor is the daily intake of cholesterol, and different dietary habits may affect the epigenetic regulatory factors of cancer development. And these ideas require more statistical analysis and experimental research to further elucidate.

## 3 Abnormal triglyceride metabolism and tumor

Previous studies have found that triglycerides are involved in reprogramming of lipid metabolism and can promote tumor progression. In a large prospective cohort consisting of 514097 participants and 38746 cancer patients, significant associations between serum triglycerides and cancer risks in the whole and multiple sites were observed, providing epidemiological evidence for the possible role of serum triglycerides in the incidence rate of cancer (43). The study observed a relatively significant positive correlation between serum triglyceride levels and male colon cancer, and an association between serum triglycerides and an increased risk of respiratory cancer in both males and females. In other prospective studies, serum triglycerides have also been observed to be associated with an increased risk of various cancers such as cervical cancer, thyroid cancer, and lung cancer (44). Basic research has found that in model mice with hepatocellular carcinoma induced by injection of diethylnitrosamine, triglycerides deposit, saturated and monounsaturated and polyunsaturated triglycerides increase, and diglycerides also accumulate in cancer tissue (9). Research has shown that the inhibition of hormone sensitive lipase (HSL) and triglyceride lipase (ATGL) can improve certain characteristics of cancer-related cachexia, thereby helping to prevent cachexia (45). Low levels of ATGL mRNA were associated with significantly reduced survival rate in patients with ovarian cancer, breast cancer, gastric cancer, and non-small cell lung cancer (46). It is worth noting that lung tumors, including invasive adenocarcinoma, spontaneously form in mice lacking ATGL, indicating that ATGL plays an important role in controlling tumor development (46). The oncogene KRAS can control the storage and utilization of lipid droplets by regulating HSL. In pancreatic cancer, HSL expression is down regulated, destruction of KRAS-HSL axis reduces lipid storage, reprogrammed tumor cell metabolism, and inhibits invasive migration of pancreatic cancer (47). Xu M et al. demonstrated that HSL deficiency is associated with adipose tissue and inflammation of the pancreas, and is involved in the progression of pancreatic cancer (48). In the triglyceride synthesis pathway, Long Chain Acyl-CoA Synthetases (ACSL) 1 promote prostate cancer progression by increasing fat production and fatty acids β-oxidation, knockout of ACSL1 inhibits the proliferation and migration of prostate cancer cells *in vitro*, as well as the growth of prostate xenografts *in vivo* (16). In breast cancer cells with HER2 positive and ACSL4 negative, the up regulation of ACSL4 will increase the proliferation rate and lead to drug resistance of lapatinib (49). In prostate cancer, androgen receptors synergistically regulate the expression of ACSL3 and ACSL4, and knocking out ACSL4 inhibits the proliferation, migration, invasion, and xenograft growth of androgen receptor dependent prostate cancer cells (50). Meanwhile, prostate tumors unrelated to the androgen receptor pathway are more dependent on ACSL4 mediated fatty acid metabolism, which may be associated with higher tumor invasiveness and metastasis. In addition, it has been determined that Carboxylesterase 1 (CES1) can promote CRC cell survival by promoting fatty acid oxidation and preventing toxic accumulation of triacylglycerol (51). Additionally, NF-κB signal enhances the activation of WNT/β-catenin in intestinal epithelial cells through CES1 dependent lipid catabolism, induces non stem cell dedifferentiation that obtains tumor initiation ability, stimulates tumor inflammation and survival of intestinal tumor cells (51), and participates in the regulation of energy metabolism, metabolic stress adaptation, and epithelial mesenchymal transition.

However, some studies have shown a lack of correlation between triglycerides and tumor risk (48). Borena W et al. speculate that low levels of ROS and oxidative stress affect their correlation. From the level of evidence, large samples and random data should be more convincing, and triglycerides are more affected by diet in the short term. Therefore, the duration of action of high triglycerides, the degree of impact on other lipid metabolism pathways, the lipid metabolism characteristics of primary tumors, and factors such as race, age, and region may all affect the observations. Therefore, more evidence is needed to demonstrate the role of triglyceride metabolism in tumor progression and reveal its mechanisms.

## 4 Abnormal sphingolipid metabolism and tumor

Sphingolipid is a kind of lipid containing the skeleton of sphingosine, which generally exists in plant and animal membranes, especially abundant in the tissues of the central nervous system. Sphingolipids include sphingomyelin, glycosphingolipids and gangliosides. Sphingolipids and their metabolites are a class of very important active molecules, which participate in numerous monumental signal transduction processes such as regulating cell growth, differentiation, aging and programmed cell death. The important role of sphingolipids in tumors has been gradually recognized in recent years.

### 4.1 Sphingolipid: ceramide and sphingosine 1-phosphate

Sphingolipid has been proved to be a key signal molecule in many pathophysiological processes such as inflammation, cancer, metabolic diseases, neurodegenerative diseases, and lysosomal storage diseases (52). Ceramide and sphingosin-1-phosphate (S1P), two central bioactive lipids of sphingolipid, play opposing roles in regulating cancer cell death and survival respectively (53).

Ceramide is synthesized through three pathways, namely, *de novo* synthesis pathway, sphingomyelin hydrolysis pathway and salvage pathway. The *de novo* synthesis route is to generate ceramide from palmitic acid and serine through a series of steps such as condensation, reduction and dehydrogenation. It is then transported to Golgi apparatus through vesicles or ceramide transporters, and then further converted into other sphingolipids, such as sphingomyelin and complex glycosphingolipids. Sphingomyelin hydrolysis pathway is that sphingomyelinase decomposes sphingomyelin in cell membrane to produce ceramide. The remedy is to utilize the circulation of sphingosine, reuse the long chain sphingosine, and reform ceramide under the catalysis of ceramide synthase (CERS). The rescue pathway also involves the circulation of exogenous short chain C2~6 ceramide to generate endogenous long chain C14~26 ceramide, which exerts a variety of signal functions in cancer cells, such as inhibiting proto oncogene protein Myc and telomerase (54).

Ceramide, as the central molecule of sphingolipids metabolism, is the precursor of all sphingolipids synthesis. Ceramide can be converted into ceramide-1-phosphate (C1P) and sphingomyelin by ceramide kinase (CERK) and sphingomyelin synthase, respectively. Ceramide can also produce sphingosine under the hydrolysis of ceramidases, and sphingosine can participate in the ceramide recovery pathway or be phosphorylated by SPHK1/SPHK2 (sphingosine kinase) to produce sphingosine 1-phosphate (S1P).

In the ceramide metabolic pathway, glucose ceramide synthase (which can decompose ceramide) is associated with the drug resistance of head and neck cancer or liver cancer cells to chemotherapy drugs. The high expression of CERS6 is closely related to the poor prognosis of ovarian cancer. Shi Y et al. have confirmed that the knockout of CERS6 inhibits the proliferation, invasion, metastasis and apoptosis of serous ovarian cancer cells, and regulates cell cycle by affecting AKT/mTOR/4EBP1 signal pathway (54). In addition, p53 regulates specific ceramide biosynthesis through direct transcriptional activation of CERS6 and induces cell death in p53 wild-type human lung cancer cells (55). The study of Chen H et al. for the first time proved that CERS6 can be used as a treatment sensitive biomarker for the chemotherapy response of triple negative breast cancer patients (56). In addition to CERS6, CERS2 (57), CERS1 (58), and CERS4 (59) have also been proven to be associated with tumor progression. Similarly, CERK/C1P pathway plays a prominent role in regulating the growth, survival and spread of cancer cells (60). Activation of CERK/C1P pathway inhibits the formation, metastasis and migration of A549 lung cancer cells (61). Rivera IG et al. also demonstrated that CERK expression/activation and C1P may be related factors regulating migration and invasion of human pancreatic cancer cells (62). The inhibition of CERK restricts AKT activation and translocation to mitochondria in mutant KRAS lung cancer, blocks phosphorylation of mitochondrial membrane potential related protein VDAC, thus affecting iron ptosis and cancer cell survival (63). In triple negative breast cancer, the up regulation of CERK is significantly related to its chemoresistance, which may be mediated by the up regulation of Ras/ERK and PI3K/Akt pathways (64). It is worth noting that CERK has become a common therapeutic target for triple positive and triple negative breast cancer cells (65). The inhibition of acid ceramidase in CDase family causes mitochondrial dysfunction and oxidative stress in pancreatic cancer cells, thus playing an anti-tumor role (66). Upregulation of acid ceramidase was also observed in prostate cancer (67). Ceramide transfer proteins also play an indispensable role in tumor progression. Ceramide transfer protein inhibits ceramide metabolism by enhancing/inhibiting epidermal growth factor receptor and lysosomal associated membrane glycoprotein 2, thereby inducing cancer cell death or reducing drug resistance (53). S1P is also a potential target for tumor therapy. Studies have shown that increased levels of S1P were detected in the SPHK2 knockout mice, which exacerbates chronic intestinal inflammation, and through S1PR1–NF-κB-IL-6 (interleukin-6)–STAT3 (activator of transcription factor 3) signal leads to the development of colitis related cancers (54). Analysis of metabonomics and network pharmacology shows that the effective anti-cancer component of Weijing Tang - tricin inhibits tumor growth primarily by inhibiting PRKCA/SPHK/S1P signal and anti-apoptotic signal (68). All these evidences indicate that sphingomyelin and ceramide occupy an important position in tumor progression.

The length of ceramide acyl chain determines its function. Long-chain species (C16-C20) increase cell death, oxidative stress and insulin resistance, cell death and oxidative stress, while derivatives of very long chain (C>22) have the contrary effect. In the model mice injected with diethylnitrosamine for hepatocellular carcinoma, most ceramide species were induced, and the ratio of long-chain and very long-chain ceramide increased (9). Haberl EM et al. confirmed that ceramide mediates the anticancer effect of different chemotherapy drugs, and restoring ceramide level can inhibit human hepatocellular carcinoma, and speculated that short chain ceramide can prevent tumor progression. However, ceramide increased in mouse tumors and decreased in human hepatocellular carcinoma tissues (69). Therefore, the model of hepatocellular carcinoma induced by diethylnitrosamine is not suitable for testing new drugs targeting neoadipogenesis or ceramide metabolism, but can be used to study how tumors escape the cytotoxicity of ceramide (9).

### 4.2 Glycosphingolipids: gangliosides

The glycosphingolipids are divided into neutral and acid glycosphingolipids (including gangliosides, glucuronic acid glycoproteins with glucuronic acid, sulfated glycan phospholipids, and phosphate glycosphingolipids) according to their electric charges. Next, gangliosides are mainly introduced.

Gangliosides are glycosphingolipids on the surface of cells, which widely exist in mammalian cell membranes. There are many types of gangliosides, among them, disialogangliosides GD2 and GD3 and O-acetylated derivatives are considered to be markers of the origin of tumor neuroectoderm. Fucosyl monosialogliosides (GM1) are also overexpressed in many cancers, especially in small cell lung cancer (70). The synthetic pathways of these tumor related gangliosides are briefly described as follows: first, the first sialic acid residues catalyzed by the GM3 synthetase ST3Gal V are transferred in Golgi bodies by lactoceramide (LacCer, Gg2Cer) to form GM3 (the precursor of a series gangliosides); then the sequential action of β4GalNAc T1 and β3Gal T4 is used to extend GM3 and form GM1; and then GM1 was α-1,2-Fucosyltransferase (FUT1 and FUT2) were further fucosylated; Alternatively, the function of GD3 synthase ST8SIA1 converts GM3 into GD3 (precursor of b series gangliosides); Finally, GD3 and GD2 can be acetylated by CASD1 sialic acid O-acetyltransferase at C9 position of sialic acid to form 9-O-acetylated GD3 (9-OAcGD3) and 9-O-acetyl GD2 (9-OAcGD2), respectively, β 4GalNAc T1 and β The impact of 3GalT4 on OAcGD3 and OAcGD2 is unclear.

Gangliosides and the enzymes responsible for ganglioside metabolism are associated with many types of cancer. For example, increased expression of GD3 synthase and/or GD2 synthase mRNA is frequently observed in cancer; the promoter region of ST8SIA1 gene has been found to be relatively overexpressed in glioblastoma, melanoma and breast cancer cell lines; GD2-positive tumors highly express OAcGD2, such as sarcoma, neuroblastoma, glioma, breast cancer and small cell lung cancer cells; A large proportion of small cell lung cancer expressed very high levels of Fuc-GM1; High levels of OAcGD3 are shown in melanoma (70). Dr. Yu Wengong and his team found that ST3GaIV promotes lung metastasis of breast cancer in mice, which proves that O-GIcNAc glycosylation of protein significantly promotes tumor occurrence and metastasis, and reveals its mechanism of action. Other relevant factors need further explanation. In addition, gangliosides also regulate various growth factor receptors (GFR), including tyrosine kinase GFR, platelet derived GFR, fibroblast GFR and epidermal GFR mediated cell growth and proliferation (52).

## 5 Lipid rafts and cancer related signal pathways

In the field of cancer, lipid rafts have been proved to be linked to several survival and proliferation related signal pathways, such as PI3K/Akt pathway and IGF system (71). Gao et al. found that the destruction of lipid raft domain by MβCD (A cholesterol chelating agent) can inhibit the phosphorylation of Akt Thr308 and Ser473 sites, and enhance the apoptosis of cancer cells (15). TRAIL death receptor not only induces apoptosis, but also promotes the migration of IGF-1R to lipid rafts, thereby activating survival signal pathways to counteract TRAIL-induced apoptosis, in MGC803 and BGC823 human gastric cancer cells. In addition, some death receptors that are essential to the apoptosis signal pathway require to be transferred to lipid rafts and/or recruited by lipid rafts, which are called CASMER (apoptosis signal molecules enriched rafts), among which CD95 is the most representative (44). It has been found that Jurkat cells in acute myeloid leukemia and acute T-cell leukemia may be induced by the recruitment and aggregation of Fas/CD95 death receptor in lipid rafts (72).

Lipid rafts have also been proved to be associated with drug resistance and cancer metastasis. Yu S et al. found that the level of cellular retinoic acid binding protein II (CRABP-II) was increased in gemcitabine resistant pancreatic ductal adenocarcinoma (PDAC) cell lines, while CRABP-II knockout PDAC cells were re sensitive to gemcitabine, and confirmed that CRABP-II caused resistance to PDAC by regulating the accumulation of cholesterol in lipid rafts (73). Lipid rafts can promote angiogenesis by regulating the secretion of VEGF and VEGFR2 signal transduction in cancer cells or promote cancer metastasis through cell adhesion receptor CD44 (72).

## 6 Lipid droplets regulate lipid metabolism and participate in the invasion of tumor

In the past, lipid droplets were considered only as an energy reservoir. Some new evidence shows that lipid droplets are dynamic and functional organelle involved in key cellular processes, such as membrane biosynthesis, lipid metabolism, cell signal transduction and inflammation (74). Research on the relationship between LDs and different cellular processes involved in cancer progression and invasion is gradually maturing, such as tumorigenicity, invasion and metastasis, and chemotherapy resistance (74). Bai R et al. found the role of lipid-Lowering related factors in pancreatic cancer through bioinformatics analysis (75). Evidence suggests that acidosis induces TGF-β activate lipid droplets by regulating CD36 and diacylglycerol acyltransferase, thus increasing uptake of exogenous free fatty acid and triglyceride synthesis, inducing lipid droplet formation and promoting partial epithelial mesenchymal transition and cancer cell metastasis and diffusion (76). The above proves that lipid droplet LDs are key drivers of increased invasiveness of cancer cells. In addition, fatty acid binding protein 5 can promote lipolysis of LDs and adipogenesis of invasive prostate cancer and breast cancer cells. There are also studies indicating that LDs accumulation may be one of the reasons for EGFR tyrosine kinase inhibitor resistance in lung cancer (77). Diacylglycerolyltransferase 1 inhibitor A922500 and neutralizing anti CD36 antibodies can prevent the formation of LDs and limit the invasion of cancer cells (76). In conclusion, on the one hand, the formation of LDs is beneficial for preventing lipotoxicity and oxidative stress, but when LDs accumulate excessively, it will cause abnormal lipid metabolism, induce metabolic Reprogramming, and promote tumor progress. This may be a compensatory imbalance that exceeds the level that normal cells can store. The possible mechanism by which LDs promote tumor development at present is that they affect the activity of TGF-β2. Secondly, further confirmation is needed to confirm that diacylglycerol transferase 1 inhibitors can prevent tumor invasion.

## 7 Cancer associated adipocytes promote tumor progression

Adipocytes are one of the main stromal cells in many tissues and are considered to play an active role in the tumor microenvironment, especially cancer associated adipocytes (CAAs). CAAs not only exist near cancer cells, but also communicate with cancer cells by releasing various factors that can mediate local and systemic effects. The crosstalk between adipocytes and cancer cells leads to phenotypic and functional changes in these two cell types, which can further promote tumor progression (78). At present, CAAs have been proved to be closely related to the progression of breast cancer (79), pancreatic cancer (80), colorectal cancer (81) and other tumors. Compared with normal adipocytes, CAAs has a series of characteristics such as fibroblast like phenotype, small size, small and dispersed fat droplets, over expression of collagen VI, low expression of adiponectin and other adipokines (82). At present, the mechanism of CAAs affecting the progression of breast cancer is relatively mature. Therefore, we take breast cancer as an example to review the role of adipocytes and CAAs in tumor development and whether other tumors share the same mechanism. Among them, the mechanisms of CAAs affecting the progression of breast cancer include adipokine regulation, metabolic Reprogramming, extracellular matrix remodeling and immune cell regulation.

During the process of adipocyte formation, it is regulated by nuclear receptors PPARγ and transcription factors C/EBPα. CCAAT enhancer binding protein C/EBPβ is involved in adipogenesis at early stage and regulates transcription of peroxisome proliferator activated receptor γ(PPARγ) together with C/EBPδ. These two transcription factors synergistically participate in the expression of genes involved in insulin sensitivity, adipogenesis, and lipolysis in differentiated and mature adipocytes, as well as the secretion factors adiponectin and leptin (79). Pre adipocyte cytokine 1 is an inhibitor of adipogenesis and its expression is reduced during adipocyte differentiation. Limited evidence suggests that tumor cells may inhibit the activation of PPARγ and C/EBPα in adipocytes through the activation of Wnt pathway, and up regulate preadipocyte factor 1, induce its Reprogramming to fibroblasts, and promote tumor development. Moreover, Parathyroid hormone related proteins derived from tumor cells can inhibit fat formation by downregulating the expression of PPARγ (83), and induce adipose tissue browning by upregulating the expression of heat producing genes such as coupling protein 1 (UCP1), inducing fat breakdown to obtain more energy from mature adipocytes, and promoting bone metastasis. Surprisingly, recent research results show that tumor cells can also be transformed into non-invasive fat cells: in the mouse breast cancer model, after combined use of PPARγ agonists rosiglitazone and MEK (mitogen activated protein kinase) inhibitor trimetinib, breast cancer cells eventually differentiated into adipocytes, invasive cancer cells growth stagnate, cell plasticity loss, inhibit the invasion and metastasis of primary tumors. The combination therapy in this study brings great hope for anti-differentiation therapy, utilizing the inherent and super strong cell plasticity of invasive cancer cells to differentiate into fat cells that no longer have plasticity through external intervention therapy, thereby inhibiting the invasion and metastasis of primary tumors.

In addition, CAAs can also secrete chemokine ligand 2 (CCL2), chemokine ligand 5 (CCL5), and interleukin-1β (IL-1β), Interleukin-6 (IL-6), tumor necrosis factor-α (TNF-α), vascular endothelial growth factor (VEGF) and leptin, which can promote the invasion and metastasis of breast cancer. Among them, CCL2 mediates chemotaxis and angiogenesis by binding to CCR2 and CCR4; Inhibition of the CCL5-CCR5 signal pathway leads to activation defects of the AKT/mTOR pathway, as well as vascular and tumor growth defects (84); IL-1β contributes to tumor angiogenesis and increases the invasiveness of breast cancer by activating p38/MAPK and phosphatidylmuscular 3 kinase (PI3K)/Akt signal pathway (85); IL-6 mainly promotes invasion, metastasis and angiogenesis of breast cancer by activating JAK/STAT3 signal pathway (86); TNF-α promotes the invasion of tumor cells by stimulating the secretion of matrix metalloproteinases and epithelial mesenchymal transformation of breast epithelial cells. In addition, TNF-α And IL-1β also stimulate the expression of VEGF. In the microenvironment of breast cancer, VEGF is indeed highly up-regulated. VEGF/VEGFR triggers the proliferation, survival, migration and angiogenesis of breast cancer cells by activating carcinogenic signal pathways, including MAPK pathway and PI3K/AKT pathway (87). Interestingly, leptin and adiponectin, as a pair of antagonists, regulate the proliferation and development of breast cancer cells by binding to leptin receptors. Pathways including estrogen receptors, JAK/STAT3, and PI3K/AKT signal pathways, as well as their regulated molecules such as VEGF/VEGFR, IL-1/IL-1R, may be involved in the aforementioned mechanisms (78). In addition, leptin can also promote the proliferation of breast cancer cells *in vitro* through steroid receptor coactivator (SRC) -1/STAT3 signal pathway (88). Adiponectin triggers the AMPK signal pathway and PI3K/AKT signal pathway by binding to adiponectin receptors AdipoR1 and AdipoR2, serving as a protective factor against tumor progression (89). To sum up, tumor cells can induce Reprogramming of fat cell metabolism through interaction with CAAs to adapt to intracellular metabolic processes to support proliferation. In order to adapt to extreme energy demands, cancer cells undergo changes in the metabolism of all large molecules such as proteins, carbohydrates, and lipids. Therefore, we also speculate that cancer cells can also use the energy in fat cells to meet their own proliferation needs by inducing metabolic rreprograming of fat cells.

It is worth noting that cancer has heterogeneity in genetic and microenvironment parameters that affect cell metabolism. The changes of tumor microenvironment reflect the heterogeneity of tumor metabolism. The degree of heterogeneity of tumors at different metabolic stages can predict specific metastatic organs. In addition to cancer cells, different metabolic compartments (such as CAAs) in the tumor microenvironment play a crucial role in this process (78). Adipocytes co cultured with tumor cells exhibited higher levels of UCP1 and monocarboxylate transporter 4 (MCT4), and decreased expression of Cav-1 (90). MCT1 is highly expressed in breast cancer. MCT1 is a marker of mitochondrial metabolism and participates in the uptake of monocarboxylate, mainly transporting lactic acid to cells (91). So, there may be a “monocarboxylic acid shuttle” to achieve energy conversion between tumors and CAAs, forming metabolic coupling. In addition, amino acids such as glutamine, glycine, serine and proline, as well as arginine and ketones, have also been shown to participate in the symbiotic metabolism of CAAs with cancer cells (78). In addition to having a direct impact on tumor cells, tumors may induce stromal adipocytes to release ATP to regulate the immune microenvironment and promote cancer progression.

The phenotype of CAAs activation has been confirmed in co culture with tumor cells from several tissue types (pancreatic cancer (80), colorectal cancer (81)) and human BCs. The mechanisms of CAAs affecting the progress of breast cancer include influencing the regulation of adipokines, secreting cytokines, metabolic Reprogramming, immune microenvironment, etc. Finally, cancer cells induce dedifferentiation and lipolysis of adipocytes to provide energy for their own growth. Takehara M et al. have confirmed that CAAs can promote the progression of pancreatic cancer and increase its malignant characteristics through the expression of serum amyloid A1 (80). However, there are currently no relevant literature reports on other tumors, and we look forward to the progress of CAAs research in other tumors.

## 8 Extracellular vesicles serve as carriers for tumor cell communication

Exosomes are the most extensively studied group among the three main subpopulations of extracellular vesicles released by mammalian cells (exosomes, microcapsules, and apoptotic vesicles, also known as Apoev) (92). Exosomes and microbubbles are released from healthy cells, while Apoev is released from apoptotic or dead cells. It can contain different components, including nucleic acids, proteins, lipids, amino acids, and metabolites, which can reflect their origin in the cell. Cancer cells usually produce more exosomes than normal cells, and the exosomes from cancer cells have a strong ability to change the local and remote microenvironment (92). Extracellular vesicles, as lipid carriers, are becoming effective mediators of intercellular communication that may contribute to tumor progression.

Extracellular proteins can alter the fate of cells released from extracellular vesicles through an autocrine pathway. For example, exosomes vesicles from chronic myeloid leukemia cells contain a cytokine TGFβ1 that binds to the TGFβ1 receptor on leukemia cells, thereby promoting tumor growth by activating ERK, AKT, and anti-apoptotic pathways in the cells (93). In addition, the accumulation of nuclear DNA in the cytoplasm will lead to cell cycle arrest or apoptosis by activating reactive oxygen species dependent DNA damage response. If exosomes can carry DNA, they support cell survival and maintain cell homeostasis. Exosomes can also mediate cell interaction and regulate microenvironment through paracrine. Exosomes from host cancer cells can activate receptors or alter miRNA or RNA expression in adjacent cancer cells, thereby altering their biological phenotype. For example, glioma cells transfer extracellular vesicles containing the oncogenic receptor EGFR VIII to adjacent glioma cells lacking this receptor, thereby activating the AKT pathway in adjacent glioma cells, giving these cells the ability to grow independently of anchoring (94). Mutated KRAS and other oncogenes such as EGFR and SRC can transfer to tumor cells through exosomes, promoting colon cancer invasion (95). Apoptosis vesicles may be related to tumor invasiveness and drug resistance. Exosomes not only metastasize between cancer cells, but also between cancer cells and stromal cells: stromal cells receive exosomes from cancer cells, producing a plasma microenvironment; conversely, cancer cells utilize extracellular vesicles released by stromal cells to promote epithelial mesenchymal transition and angiogenesis, promoting cancer cell proliferation or invasion.

In addition, exosomes play an important role in the dedifferentiation process of adipocytes. Wu Q et al. have proved that the physiological purpose of miRNA-144, miRNA-126, miRNA-155 and other special tumor miRNAs transfer from breast cells to fat cells in the microenvironment of breast cancer through exosomes, leading to fat cells transforming into CAAs (90) to produce exosomes is still largely unknown, and further research is needed. One speculated effect is that exosomes may remove excess or unnecessary components from cells to maintain intracellular homeostasis. The identification and isolation of single exosomes, as well as cryoelectron microscopy analysis, may greatly enhance our understanding of the basic biology of exosomes and their applications in applied science and technology (96). This knowledge will provide information on the potential of exosomes for treating various diseases, including cancer and neurodegenerative diseases.

## 9 Lipid metabolism is closely related to tumor drug resistance

Chemotherapy and immunotherapy are the main treatment methods for metastatic cancer. Common tumor chemotherapy drugs include antibiotics, plant-based drugs, platinum drugs, etc. However, drug resistance limits its effectiveness and leads to a significant reduction in treatment effectiveness. Cancer chemotherapy and immunotherapy resistance have complex multifactorial mechanisms, among which lipid metabolism plays a crucial role. Abnormal lipid metabolism interferes with the normal regulation of cells and interferes with the effectiveness of immunotherapy and chemotherapy drugs.

### 9.1 Lipid metabolism and chemotherapy

In the process of cholesterol and triglyceride metabolism, cholesterol itself, as the end point and starting point of lipid metabolism, is related to the progress, invasion and drug resistance of breast cancer, non-small cell lung cancer (97) and other cancers (98). Metabolic enzymes can also affect tumor resistance by increasing metabolic intermediates. Fatty acid synthase is a key enzyme in the fat formation pathway, and its overexpression leads to cancer resistance to genotoxicity drugs by increasing DNA repair. The HMGCR inhibitor simvastatin can also enhance the efficacy of capecitabine chemotherapy by inhibiting NF-kB regulated markers of proliferation, invasion, angiogenesis, and metastasis (99). In addition, the expression of metastasis associated genes 1, acetyl CoA synthase 2 (100), stearyl CoA desaturase, and 12 lipoxygenase (101) in human colon cancer can enhance chemotherapy resistance to platinum drugs or paclitaxel by regulating lipid metabolism (99, 102). Some transporters, such as ABC transporters, have been identified to be associated with multiple drug resistance, resulting in resistance of cancer cells to 5-FU, vincristine, cisplatin and other chemotherapy drugs (103). In addition, cholesterol regulatory factors such as SREBP2 can be induced and activated by Caspase-3 (cysteine enzyme 3) to promote cholesterol biosynthesis, thus leading to resistance to targeted antiangiogenic drugs, including sorafenib or lovatinib, by driving the activation of Hedgehog signal pathway (104). In addition to platinum and plant-based chemotherapy drugs, antibiotic drugs such as penicillin can make cells re sensitive to oxaliplatin by inhibiting FAO and promoting intracellular ROS accumulation (105). Other chemotherapy drugs, such as doxorubicin, lower the rate limiting enzyme HMGCR levels of cholesterol synthesis (106). On the contrary, cyclophosphamide has no effect on blood lipids. These data indicate that the different effects of chemotherapy drugs on lipid metabolism may also be related to drug efficacy. Multiple types of chemotherapy drugs and targeted anti angiogenic drugs inevitably exhibit resistance, and targeted lipid metabolism may become a new approach to address resistance at present.

During sphingolipid metabolism, SPHK1 is a lipid kinase that phosphorylates sphingosine to generate S1P. S1P has been proved to be positively correlated with chemotherapy resistance of breast cancer, colorectal cancer and non-small cell lung cancer (107). The study of Qin Z et al. showed that SPHK1 was positively related to the cisplatin resistance of bladder cancer cells, and showed anti apoptotic effect by down-regulation of apoptosis related protein and up-regulation of anti-apoptotic protein (Bcl-2) in overexpression cell lines (107). Its potential mechanism of inducing cisplatin resistance and apoptosis inhibition may be to activate STAT3 by binding to the octamer containing non POU (with highly conserved DNA binding domain) domain, and become a potential new target for bladder cancer treatment. Many evidences show that activated SphK1 is involved in tumor genesis and resistance. Ceramide and sphingosine metabolic enzyme inhibitors and synthetic ceramide can be used as sensitizers for radiotherapy and chemotherapy of head and neck squamous cell carcinoma (108). Phosphatidylcholine has been identified as significantly associated with tumor resistance. Phosphatidylcholine is hydrolyzed by phospholipase A2 to produce lysophosphatidylcholine and arachidonic acid (AA), and COX-2 catalyzes the conversion of AA to prostaglandin E2. Liao et al. reported that when cytosolic phospholipase A2α is targeted, the Ras/MEK/ERK and Akt/b catenin signal pathways are inhibited, ultimately enhancing the effectiveness of chemotherapy (109). Hemolytic phosphatidylcholine acyltransferase 1 converts lysophosphatidylcholine into phosphatidylcholine in the presence of acyl coenzyme a. In gastric cancer, the expression of lysophosphatidyl acyltransferase 1 is significantly up-regulated, which promotes the proliferation of cancer cells, improves the survival period, and enhances the ability of cell migration of cancer (110). Chen et al. demonstrated that artificial inhibition of phospholipase A2 can increase the chemical sensitivity of gastric cancer cells to 5-Fu (111).

The regulation and epigenetics of adipocytes, lipid droplets, and ester rafts also play an important role in tumor resistance. Studies have shown that pancreatic cancer cells can induce adipocytes to dedifferentiate into CAA. CAA promotes the malignant characteristics of cancer through the expression of SAA1, suggesting that SAA1 is a new therapeutic target for cancer. Moreover, extracellular vesicles derived from adipocytes can reduce the susceptibility to ferroptosis in CRC and mediate resistance to oxaliplatin. In addition, the accumulation of LDs drives the death resistance of CRC cells to 5-fluorouracil and oxaliplatin treatment (112). Keratin 6 can enhance resistance to cisplatin by regulating the formation of lipid rafts (113). Interestingly, epigenetics is also associated with tumor chemotherapy resistance, for example hypermethylation of miR-129-5p induces resistance of gastric cancer cells to chemotherapy drugs such as 5-FU, vincristine, and cisplatin (100).

It is worth noting that lipid metabolism also affects chemotherapy resistance by changing the immune microenvironment. Mesenchymal stromal cells (MSCs) in the tumor microenvironment are considered as important factors to promote the development of chemotherapy resistance. As an important component of the tumor environment, MSCs can promote chemotherapy resistance and help cancer cells overcome the anticancer effects of chemotherapy drugs by secreting protective cytokines, even product gene mutations and alter transcriptional expression. In gastric cancer, MSCs can promote self-renewal and chemotherapy resistance of tumor cells through fatty acid oxidation (FAO). And the FAO inhibitor Etomoxiry can significantly reverse stem cell characteristics and resistance to 5-FU and oxaliplatin (114). Interestingly, an existing study shows that co culture of MSCs with gastric cancer cells activates FAO in gastric cancer cells, thereby enhancing chemotherapy resistance (115). In addition, MSCs can also up regulate non-coding RNA AGAP2-AS1 to affect the expression of CPT1 (carnitine palmitoyltransferase 1), thereby promoting FAO and chemotherapy resistance of breast cancer (116). These results indicate that tumor cells can interact with mesenchymal stem cells to promote tumor cell metabolic reprogramming and drug resistance.

### 9.2 Lipid metabolism and immunotherapy

Immunotherapy is currently an important method for tumor treatment. At present, the most widely used immunotherapy drugs are blocking antibodies targeting immunosuppressive receptors, such as PD-1 (Programmed Cell Death Protein-1), PD-L1 (Programmed Death Ligand-1), CTLA-4 (Cytotoxic T Lymphocyte Associated Protein 4), etc. (117). In recent years, compared to CTLA-4, anti PD-1 antibodies have shown stronger clinical efficacy and superior tolerance. However, powerful as anti-PD-1 antibody also inevitably develop resistance. The mechanism of anti PD-1 antibody resistance is still unclear, which may be related to TYRO3 (118), KRAS (119) and other gene mutations, and the mutation rate of the tumor itself (117), β-catenin related immune escape (120), the expression level of PD-1 in the tumor itself (117) and IFN-γ related to the disruption of tumor signal pathways. Due to the presence of drug resistance, the response rate of many tumors to immune checkpoint inhibitors is only between 20% -40% (121). So, it is very important to address the resistance of immune checkpoint inhibitors. At the same time, targeted lipid metabolism demonstrates the ability to enhance the efficacy of immunotherapy. In tumor immunotherapy, LXR activation therapy produces a strong anti-tumor response in mice and enhances T cell activation in various immunotherapy studies, suggesting that the lipid metabolism regulatory factor LXR may be a target for improving the efficacy of tumor immunotherapy (15). In addition, the expression of key lipogenic enzymes such as acetyl CoA carboxylase is significantly negatively correlated with the infiltration level of CD8+T cells and immune cell lysis activity in gastric cancer, indicating that inhibiting acetyl CoA carboxylase can enhance the anti-tumor immunity of gastric cancer (122). Although there is currently no clinical study on the combination of immune checkpoint inhibitors and targeted lipid metabolism drugs, targeted lipid metabolism is likely to bring more significant clinical efficacy to immunotherapy. Further exploration is needed on the relationship between lipid metabolism and immunotherapy resistance.

The above indicates that the metabolism of cholesterol, phospholipids, triglycerides, lipid rafts, lipid droplet formation, and epigenetics play an indispensable role in the development of tumor resistance. Lipid metabolism is closely related to tumor chemotherapy and immunotherapy resistance, including lipid regulatory factors, lipid metabolism related enzymes, and lipid metabolism intermediates. Some studies have shown that regulating lipid metabolism can enhance the effectiveness of chemotherapy and immunotherapy, so targeted lipid metabolism combined with chemotherapy is expected to become a critical method to address tumor resistance.

## 10 Potential role of lipid metabolism related drugs in tumors

### 10.1 Statins

Statins are a cholesterol lowering drug that selectively inhibits HMGCR, and their anti-tumor properties are receiving increasing attention. Statins inhibit the development of cancer cells by blocking the valproate pathway and damaging the YAP/TAZ (the main transcription regulator of normal organ growth and tumor growth) dependent transcriptional response (14). Statins including pivastatin (123), fluvastatin (15) and simvastatin (124) have been proved to be effective in inhibiting tumor cell proliferation and angiogenesis and promoting cell apoptosis. Its possible mechanisms include: inhibiting Akt and Braf/MEK/ERK1/2 pathways to prevent cell growth and promote cell apoptosis (15); Pre acylated Ras/Raf/MEK and PI3K/Akt/mTOR signal pathways inhibit tumor endothelial cell growth (123); Increase the expression of miR-140-5p, thereby inhibiting proliferation and inducing apoptosis (12). It is worth noting that Hu T et al. found that pivastatin is also effective in the chemotherapy resistance of lung cancer cells (123). A clinical trial for breast cancer patients showed that they received high-dose atorvastatin (80mg/day) 2 weeks before surgery. This treatment reduced the proliferation of breast cancer by affecting the expression of cyclin D1 and p27 (125). In addition, statins can also manipulate lipid rafts to affect tumor development by lowering cholesterol levels. The consumption of cholesterol in the lipid raft of non-small cell lung cancer can inhibit the phosphorylation of lipid raft related non receptor tyrosine kinase Src and the dislocation of local adhesion complex of lipid raft (14), which is related to tumor formation, cell migration and invasion. Statins can also induce autophagy, iron death, and affect the tumor microenvironment to play an anti-tumor role, but the specific mechanism is still questionable (125).

Compared with hydrophilic statins (such as pravastatin and rosuvastatin), lipophilic statins (such as simvastatin) show greater ability to penetrate cell membranes and passively diffuse into hepatocytes and non-hepatocytes, as well as higher apoptosis promoting activity (126). Due to its high cytotoxic potential, lipophilic statins may be beneficial for cancer treatment. Lipophilic statins act as EMT signaling pathway antagonists in breast cancer stem like cells by inhibiting the mevalonate pathway (125).

In summary, statins can affect tumor progression through various factors, such as tumor cell proliferation, angiogenesis and apoptosis, as well as chemotherapy resistance. However, in clinical studies, statins have not shown any inhibitory effect on tumors. We collected clinical studies on statins and tumor progression from Pubmed, including all randomized controlled trial studies (**Table 1**), and collected some information to better understand the current research status of statins in tumors.

**Table 1:** Summary of RCT studies on statins and tumor progression.

First author, Year	Country	Characteristics of participants	Intervention dose	Number of participants, n		Mean age, year		Male, n		Follow-up time	Outcome index	Outcome
				Statin	control	Statin	control	Statin	control			
Kawata S 2001	Japan	Unresectable hepatocellular carcinoma	Pravastatin, 40mg	41	42	62	62	31	34	16.5 ± 9.8 months	MST	Pravastatin prolongs the survival of patients with advanced HCC.
Timo E Strandberg 2004	Denmark, Finland, Iceland, Norway, Sweden	Simvastatin treatment for 5 years, followed by open label statin treatment	–	414	468	–	–	–	–	10 years	TC, HDL, LDL, cancer mortality and incidence rate	There was no difference in cancer mortality and incidence rate between the original simvastatin group and the placebo group.
Heart Protection Study Collaborative Group 2005	Britain	with vascular disease or diabetes	Simvastatin, 40mg	814	803	40-80	40-80	653	780	5 years	TC, HDL,cancer mortality and incidence rate	There is also no significant evidence that cholesterol lowering therapy has an adverse effect on the incidence rate of cancer.
Hannah Graf 2008	–	Patients with advanced liver cancer	Pravastatin, 20-40mg	52	131	66 ± 10	63 ± 10	41	108	3 years	MST, mortality rate, the incidence of perihepatic lymph node enlargement	Compared with chemoembolization alone, chemoembolization combined with pravastatin can improve the survival of patients with advanced liver cancer.
Carol F. 2010	America	Women with histologically confirmed DCIS or stage I breast cancer	Fluvastatin, (80mg/20mg)	20	20	55	55	0	0	28 (21-50) days	Ki-67, cystatin 3, MRI tumor volume, CRP, TC	Fluvastatin showed measurable biological changes in high-grade stage 0/1 breast cancer by reducing tumor proliferation and increasing apoptosis activity.
Inge R.H.M. Konings 2010	–	Patients with advanced gastric cancer	Pravastatin, 40mg	15	15	59 (36-73)	57 (42-74)	11	13	6 months	PFS-6,RR,PFS,OS	Adding pravastatin to ECC does not improve the prognosis of patients with advanced gastric cancer.
Ji-Youn Han 2011	America	Patients with advanced non-small cell lung cancer	Simvastatin, 40mg	52	54	58 (20-76)	60 (32-84)	25	29	28 years	RR,PFS,OS	Simvastatin has not shown any benefits in patients with non-small cell lung cancer. However, simvastatin combined with gefitinib showed improved efficacy in wild-type EGFR non adenocarcinoma patients.
Paul J. Limburg 2011	America	Subjects with multiple/advanced colon adenomas or previously resected colon cancer	Atorvastatin, 20 mg	22	22	61 (44-78)	57 (44-72)	56	15	6 months	The percentage change in the number of ACFs in the rectum within the arm (%) Δ ACF),Ki67, Cystatin-3	Atorvastatin does not reduce the risk of colon cancer.
JungYong Hong 2014	–	Patients with advanced pancreatic cancer	Simvastatin, 40 mg	58	56	60 (38–80)	56 (25–74)	36	33	3 weeks	TTP,1-year expected survival rate,overall disease control rate	Gemcitabine combined with low-dose simvastatin in the treatment of advanced pancreatic cancer does not provide clinical benefits, but does not lead to increased toxicity.
Seung Tae Kim 2014	Korea	Advanced gastric cancer	Simvastatin, 40mg	120	124	53.5 (20-78)	54.5 (24-79)	91	85	65 months	FPS,RR,OS	Simvastatin does not alter FPS in patients with advanced gastric cancer
KennethG. Linden 2014	–	Atypical nevus (considered as precursor of melanoma)	Lovastatin, 40mg, and increased to 80mg at week 6	34	32	42.8 (10.96)	42.2 (11.28)	12	12	6 months	Atypical level of histopathology,TC,LDL, number of moles	Compared with placebo, lovastatin had no benefit in reducing the primary endpoint of histopathology atypia, any secondary endpoint of clinical atypia, the impact on nevus number, and any significant changes in molecular biomarkers.
YongLi Ji 2016	America	Premenopausal patients with breast cancer	Atorvastatin, 40mg	31	32	43.7 (4.1)	43 (4.5)	0	0	1 years	TC,LDL,MD,IGF-1	Atorvastatin has no significant effect on MD.
Youngjoo Lee 2017	Korea	Patients with advanced NSCLC	Simvastatin, 40mg	36	32	59 (44–80)	67 (44–78)	32	27	48 weeks	RR,OS,PFS, ORR	There is no significant difference in efficacy between the two groups.
Michael J. Seckl, 2017	Britain	Confirmed small cell lung cancer patients	Pravastatin, 40 mg	422	424	64 (41-86)	63 (42-85)	219	214	Median: 39.6 months	OS,PFS,RR	Although pravastatin 40mg combined with standard small cell lung cancer is safe, it is not beneficial to patients.
Teemu J Murtola 2018	Finland	Prostate cancer patients	Atorvastatin, 80mg	80	78	64 (59–68)	63 (58–68)	80	78	Median: 27 days	Ki-67,PSA	Compared to placebo, cholesterol lowering atorvastatin generally does not reduce the proliferation rate of prostate cancer.
Jouve JL 2019	France	Child-Pugh A advanced HCC patients receiving early systemic treatment	Pravastatin, 40mg	162	161	68 (37-86)	68 (39-85)	156	142	35 months	OS,PFS,TC,TG,TTP	The combination of sorafenib and pravastatin did not improve the overall survival rate of the study population.
Alexandre L 2020	Britain	Esophageal cancer patients who have undergone esophagectomy	Simvastatin, 40mg	16	16	66.6 (8.7)	62.7 (12.3)	13	12	1 year	LDL,OS,PFS,death rate	There is no significant difference between the two groups.
Altwairgi AK 2021	Saudi Arabia	Combined glioblastoma Standard treatment	Atorvastatin 40mg for 3 weeks, followed by 80mg daily	36	65	52 (20–69)	47 (18–81)	22	49	19 months	PFS-6	Atorvastatin did not show any improvement in PFS-6.
Ghafarzadeh M 2021	–	fibroid	Atorvastatin, 20mg	45	45	40.35 ± 3.32	39.63 ± 36.3	45	45	3 months	Fibroma size	This study suggests that atorvastatin treatment may actively reduce the size of fibroids, and this decrease is only statistically significant in the first month.
Erwin Danil Yulian 2021	Indonesia	Patients with locally advanced breast cancer	Simvastatin, 40mg	30	30	49.4 (36-66)	46.9 (28-65)	30	30	17 months	ORR,CR	In locally advanced breast cancer patients with HER2 overexpression, 40 mg simvastatin can improve the efficacy of FAC.
Jeong IG 2021	Korea	Patients with high-risk pathological characteristics after radical prostatectomy	Atorvastatin, 20mg	183	181	65.3 ± 7.3	65.1± 7.1	183	181	1 year	TC LDL PSA Prostate cancer recurrence rate	Adjuvant use of atorvastatin for 1 year is not associated with reduced risk of disease recurrence.
Karkeet RM 2022	Egypt	Castrated mPC patients undergoing castration treatment	Rosuvastatin, 20mg	40	30	>50	>50	40	30	6 months	PSA,EGFR, CAV1,LDL, HDL,TG,TC, AKR1,HMGCR,A1 ABCA1, SLDLRP1	The peripheral lipid-lowering effect of rosuvastatin may affect the treatment results and survival rate of castrated mPC patients.
Wilson, B. E 2022	–	Metastatic castration resistant prostate cancer	Statin	Statin A 236	Control A 103	–	–	100%	100%	–	OS	The use of statins in Group A was associated with longer median OS, while Group B had the opposite result.
				Statin B 436	Control B 229							
Erwin Danil Yulian 2023	Indonesia	Patients with locally advanced breast cancer	Simvastatin, 40mg	33	33	48.13 (28-66)	48.13 (28-66)	33	33	17 months	Ki-67,Tumor size	There was no statistically significant difference in tumor reduction between the two groups, but the expression of HMGCR was associated with tumor characteristics with lower invasiveness.

PSA prostate specific antigen; EGFR epidermal growth factor receptor; NSCLC non-small cell lung cancer; OS total survival time; PFS disease-free survival period; Median survival time of MST; CAV1 Caveolin-1; TG triglycerides; TC total cholesterol; LDL low-density lipoprotein cholesterol; HDL high-density lipoprotein cholesterol; AKR1 aldehyde ketone reductase; HMGCR HMG-CoA Reductase; ABCA1 ATP binding cassette transporter A1; SLDLRP1 soluble low-density lipoprotein receptor associated protein 1; PFS-6 progression free survival at 6 months; TTP disease progression time; RR efficiency; Objective response rate of ORR; CR complete response rate; MD breast X-ray density; IGF-1 insulin growth factor 1; TG triglycerides.

### 10.2 Fibrates

Yamasaki et al. demonstrated that beta drugs can reduce the proliferation of cancer cells and promote cell apoptosis (127). Some studies have shown that fenofibrate can induce p53 protein accumulation and reprogram the tumor’s immune microenvironment in HPV positive head and neck squamous cell carcinoma (128). Chen L et al. showed that fenofibrate showed antitumor activity *in vitro* and *in vivo* through mitochondrial and metabolic Reprogramming (129). In addition, fenofibrate also inhibits mTOR-p70S6K signal and induces cell death in human prostate cancer cells (130). However, a systematic review and meta-analysis of 17 long-term randomized placebo-controlled trials in 2012 showed that the impact of beta drugs on cancer was neutral (131). Therefore, higher quality research or larger scale experimental studies are needed to demonstrate the correlation between beta drugs and tumors.

### 10.3 Others

The precursor invertase Bacillus subtilisin/Coxin 9 (PCSK9) could regulate cholesterol metabolism by connecting LDLR, and reduce cholesterol circulation by targeting lysosomes to destroy receptors (132). Inhibition of PCSK9 by gene deletion or PCSK9 antibody can increase the expression of major histocompatibility protein class I protein on the surface of tumor cells, and promote the strong tumor invasion of cytotoxic T cells (133). However, the relationship between PCSK9 and tumor needs further proof.

A study on pancreatic and glioma cancer cells showed that methyl-β-Cyclodextrin depletes cholesterol in cell membranes and destroy lipid rafts, leading to an increase in CD44 extracellular lipid distribution, and promoting CD44 related tumor metastasis mediated by ADAM-10, a metalloproteinase (72). methyl-β-Cyclodextrin can also inhibit cell growth and cell cycle arrest through the expression of cyclin A and D1 mediated by prostaglandin E (2) independent pathway (134), and inhibit endothelial adhesion of monocytes induced by lipopolysaccharide or oxidized low-density lipoprotein (135). The above indicates methyl β-Cyclodextrin may become a new drug for tumor therapy.

As a cholesterol absorption inhibitor, studies have also found that ezetimibe can demonstrate its anti-tumor effect by inhibiting angiogenesis (15).

Acid sphingomyelinase (SMase) causes sphingomyelin to hydrolyze into ceramide, thereby inducing apoptosis of p53 defective glioblastoma cells (136). Overexpression of acidic SMase makes glioma cells sensitive to chemotherapy, gemcitabine and doxorubicin (137). The activation of neutral SMase in C6 glioma cells is believed to increase the activation of mitogen activated protein kinase through the up regulation of ceramide, thus leading to apoptosis (136). Sphingomyelinase inhibitors may contribute to tumor therapy. In addition, the development of small molecule drugs that inhibit the activity of acid ceramidase is a promising method to improve the standard cancer treatment and clinical results of patients (67).

In addition, nanoparticle mediated CERK siRNA delivery and hydrogel mediated continuous delivery of CERK inhibitors to tumor sites can also inhibit tumor progression (65).

It is worth noting that the mRNA in the exosomes can be transferred to the receptor cells and play a role in the receptor cells (92). Therefore, exosomes can be designed to deliver different therapeutic payloads, including short interfering RNA, antisense oligonucleotide, chemotherapy drugs and immunomodulators, and have the ability to deliver them to the desired target. The lipid and protein composition of exosomes can affect their pharmacokinetic properties, and their natural components may play a role in improving bioavailability and reducing adverse reactions (96).

Epigenetic related demethylation and histone modification drugs are also new anticancer therapies. Wang B et al. believe that the dexcitabine based regimen can serve as an alternative first-line chemotherapy regimen for elderly patients with acute myeloid leukemia (138). And it shows anti-tumor activity in prostate cancer and non-small cell lung cancer. The possible mechanism is that the silencing of tumor suppressor genes induced by DNA methylation is relieved.

In addition, viruses are also used to combat tumor cells. Oncolytic virus is a kind of virus that can specifically infect tumor cells and induce their death. But at present, only one oncolytic virus - genetically modified herpesvirus T-VEC has been approved by the US FDA for the treatment of melanoma.

From radiochemotherapy drugs to targeted therapy to immunotherapy, as well as a variety of tumor treatment methods including interference RNA and oncolytic virus, human beings have made an unprecedented attempt to defeat tumors. Although drug resistance has always been a problem, targeted lipid metabolism is still expected to become a promising anti-tumor strategy.

## 11 Summary

Abnormal lipid metabolism is closely related to tumor occurrence, and intermediate products of lipid metabolism, including lipid metabolism rate limiting enzymes and various lipid regulatory factors, play important roles in tumor occurrence, development, invasion, and metastasis. And lipid rafts, lipid droplets, adipocytes, and exosomes related to lipid metabolism are also involved in the development of tumor resistance. This article summarizes the current research status of carcinogenic signal pathways and major lipid regulatory factors involved in lipid metabolism regulation, as well as cholesterol, triglycerides, and sphingolipids metabolism pathways and tumor progression. In addition, this article emphasizes the important role of epigenetics in promoting tumor progression through lipid metabolism. Finally, this article introduces the involvement of lipid metabolism in the development of drug resistance in tumor chemotherapy and immunotherapy, indicating the limitations of current tumor treatment. Due to the fact that targeted lipid metabolism can enhance the effectiveness of tumor chemotherapy and immunotherapy, targeted lipid metabolism or combination chemotherapy may bring more significant clinical efficacy to tumor treatment.

## Author contributions

CL: Literature search, Data analysis, Paper writing and Paper submission. FW: Literature search and Data analysis. SL: Literature search. LC: Literature search. JZ and LL: Critical revision and Paper submission. All authors contributed to the article and approved the submitted version.
